# Cytoplasmic Male Sterility in Sugar Beet: Mitochondrial Minisatellite and Chloroplast CAPS Markers and Field Phenotype Stability Study

**DOI:** 10.3390/life16091413

**Published:** 2026-08-26

**Authors:** Raushan Yerzhebayeva, Assel Jenisbayeva, Alfiya Abekova, Natalia Kovalchuk, Sholpan Bastaubayeva, Aigul Amangeldiyeva, Laila Tabynbayeva

**Affiliations:** 1 Kazakh Research Institute of Agriculture and Plant Growing, Almalybak 040909, Kazakhstan; jenisbayeva@gmail.com (A.J.); aabekova@mail.ru (A.A.); aigul_seidinabiyeva@inbox.ru (A.A.); tabynbaeva.lyaylya@mail.ru (L.T.); 2 Institute of Bioenergy Crops and Sugar Beets, 03100 Kyiv, Ukraine; natalakovalcuk461@gmail.com

**Keywords:** sugar beet, cytoplasmic male sterility, chloroplast genome, mitochondrial genome, minisatellite, tandem repeats

## Abstract

Cytoplasmic male sterility (CMS) is a key cornerstone of hybrid breeding in sugar beet (*Beta vulgaris* L.). Reliable identification of cytoplasmic types and evaluation of their phenotypic stability are therefore important for the effective use of CMS in breeding programs. The aim of this study was to comprehensively characterize 30 sugar beet breeding lines of different origins using molecular and phenotypic approaches. Mitochondrial minisatellite markers TR1–TR3 and the CAPS marker in chloroplast region *petG*-*psbE* were employed, together with evaluation of phenotypes for anther morphology and pollen fertility over three years under the conditions of southeastern Kazakhstan. Based on the molecular analysis, most CMS breeding lines were classified as carriers of mitotype min04, the O-type control lines with mitotype min18, and alloplasmic breeding lines with min06, which indicated the presence of an alternative origin of CMS. Combined analysis of mitochondrial and chloroplast markers revealed cytoplasm heterogeneity in several accessions, confirming the need for individual molecular control during the maintenance of CMS breeding lines. The evaluation of phenotypes over three years demonstrated that anther dehiscence and pollen fertility varied significantly depending on hydrothermal conditions. Combining molecular study with multi-year evaluations of phenotypes enables reliable cytoplasm type identification, cryptic heterogeneity detection, and the selection of breeding lines with stable male sterility for breeding of sugar beet hybrids.

## 1. Introduction

Modern sugar beet improvement relies on hybrid breeding programs aimed at developing high-yielding, stress-tolerant diploid hybrids [1]. The key mechanism for hybrid seed production is cytoplasmic male sterility (CMS), which prevents self-pollination and ensures the production of heterozygous hybrids. Commercial production of sugar beet seeds is based on Owen-type sterile cytoplasm (S-type), in which male sterility is expressed through interaction with recessive alleles at nuclear restorer-of-fertility loci [2]. The genetic basis of CMS in sugar beet is associated with rearrangements in the mitochondrial genome, leading to the emergence of aberrant mitochondrial genes that disrupt normal pollen development [3].

The molecular basis of CMS in sugar beet is associated with structural rearrangements of the mitochondrial genome and the formation of CMS-associated open reading frames (ORFs) and chimeric genes. In Owen-type CMS, the 5′ leader sequence of the mitochondrial *atp6* gene, known as *preSatp6*, encodes a CMS-associated protein. This protein accumulates specifically in sterile mitochondria and is involved in the disruption of normal pollen development [4,5]. In contrast, orf129 is associated with a different CMS type, which was found in wild *Beta* cytoplasm, particularly the E-type/I-12CMS(3) cytoplasm. Therefore, its role should be considered separately from the Owen-type CMS mechanism [6]. These differences in mitochondrial genome organization provide a molecular basis for distinguishing cytoplasmic types and have supported the development of mitochondrial markers for CMS characterization. In particular, the TR1–TR4 mitochondrial DNA markers described by Nishizawa et al. (2000) [3] have been widely used to characterize mitochondrial polymorphism and differentiate mitochondrial cytotypes, including CMS-associated and normal cytoplasms [7,8,9]. These markers represent four polymorphic tandem-repeat (VNTR/minisatellite) loci of the beet mitochondrial genome, in which variation in repeat number provides informative patterns for mitochondrial cytotype discrimination. In addition, a chloroplast CAPS marker based on the polymorphic *Hin*dIII restriction site in the *petG*-*psbE* region has been reported to be associated with Owen-type sterile cytoplasm [10]. This chloroplast CAPS marker provides an independent means of identifying the plastid component associated with Owen cytoplasm. Thus, the mitochondrial TR1–TR4 markers and the chloroplast *petG*-*psbE* CAPS marker allow for the characterization of both organellar genomes. Their combined use may reveal differences in cytoplasmic origin that cannot be detected with a single marker system. These molecular markers are therefore valuable for the identification and characterization of CMS sources in sugar beet breeding.

However, the genetic basis of Owen-type cytoplasm is characterized by limited diversity [11], which carries a greater risk of hybrid vulnerability to biotic and abiotic stresses and narrows opportunities for further breeding [12]. In this regard, the targeted expansion of sugar beet breeding gene pools through the incorporation of genetic material from wild relative species is of particular relevance [12,13]. Wild species of the genus *Beta*, such as sea beet (*Beta vulgaris* ssp. *maritima*) and *Beta patula*, serve as natural ‘reservoirs’ of genetic diversity. These species provide valuable traits, including alternative CMS types, resistance to diseases and pests, and adaptation to stress conditions [14]. Gene introgression from wild relatives into the genome of cultivated sugar beet opens new opportunities for developing hybrids with improved agronomically valuable traits and for broadening the genetic base of this crop [15].

CMS stability is a critical parameter for hybrid breeding to prevent self-pollination of maternal lines that will reduce hybrid seed quality [16,17]. The data available in the literature indicates that CMS in sugar beet may be sensitive to a range of environmental factors, including temperature stress and moisture deficit [16,18]. It was reported that exposure of CMS plants to elevated temperatures during the flowering period induced the restoration of fertility, particularly in certain genotypes. Therefore, three phenotypic groups were distinguished: stable sterile lines, stable fertile lines, and thermosensitive lines. In the latter case, plants are sterile under mild conditions, but they become fertile at elevated temperatures [18].

Despite significant progress in understanding the molecular mechanisms of CMS [19,20], the influence of climatic factors on CMS stability in sugar beet lines grown in Kazakhstan remains poorly understood. In Kazakhstan, sugar beet is cultivated in the southeastern part of the country [21,22]. The continental climate in the region is characterized by hot summers and considerable interannual fluctuations in temperature and moisture [23] that can substantially affect the phenotypic stability of CMS lines. Moreover, most studies on thermosensitive CMS have been conducted in controlled conditions [18], and data for field conditions with natural temperature fluctuations remain limited.

The aim of this study was to comprehensively characterize 30 sugar beet CMS breeding lines of diverse origin using mitochondrial TR1–TR3 minisatellite markers and the chloroplast *petG*-*psbE* CAPS marker, together with a three-year evaluation of anther morphology and pollen fertility in southeastern Kazakhstan. The study addressed whether the breeding lines differed in cytoplasmic composition and whether the identified cytoplasmic variation was associated with the expression and stability of CMS phenotypes across growing seasons.

## 2. Materials and Methods

### 2.1. Plant Materials

The studied accessions included 30 cytoplasm male-sterile (CMS) sugar beet breeding lines that were received from the germplasm collection of the Kazakh Research Institute of Agriculture and Plant Growing (KazRIAPG), the Institute of Bioenergetic Crops and Sugar Beet (Ukraine), the Kutnowski Sugar Beet Breeding Station (Poland), the A.L. Mazlumov All-Russian Research Institute of Sugar Beet and Sugar (Russia), and the Experimental Research Station of Sugar Beet (Belarus) (Supplementary Table S1).

Two CMS lines with known S-type cytoplasm were used as positive controls: PI 594911 (FC 721 CMS), obtained from the Western Regional PI Station, Washington State University (USA), and FMS-1-FD, from Florimond Desprez (France). Two O-type breeding lines with known N-type cytoplasm were used as controls: PI 632251 (FC 724), from the Western Regional PI Station, Washington State University (USA), and RF 2093, from the A.L. Mazlumov All-Russian Research Institute of Sugar Beet and Sugar (Russia).

### 2.2. Field Growing Conditions

Field experiments were conducted at the research site of the Sugar Beet Breeding Laboratory, KazRIAPG, Almaty Region, Kazakhstan (43.234102° N, 76.689032° E). Seeds of the sugar beet lines were sown in field trials at the beginning of April. Sowing was carried out in rows, 5 m long, with 10 seeds per linear meter and 50 cm between rows. Sugar beet roots were harvested in October and stored in a cold chamber at +2 to +4 °C over the winter period (5 months). To induce the formation of flowering shoots, roots were replanted at the beginning of April in the following year, and roots were arranged in rows of five per plot, 1 m in length. Observations of flowering shoots were carried out during all three consecutive years (2024–2026).

### 2.3. Meteorological and Soil Conditions of the Experimental Site

The climate of the study area corresponds to type Dfa (humid continental climate with hot summers), according to the Köppen–Geiger classification [24]. The soils are light chestnut, with a low humus content in the arable layer (1.6–1.9%). The soil is slightly alkaline with a pH of 7.8, and the clay particle content is 34.9% [25].

Meteorological data, including precipitation and air temperature, were recorded by an automatic iMetos weather station (IMT300USW, Pessl Instruments, Weiz, Austria) located 500 m from the experimental field. Mean monthly air temperature and precipitation values for the study period are presented in Table S2.

Weather conditions during the study period differed substantially in moisture availability. The hydrothermal coefficient (HTC) was used to characterize growing conditions and the moisture–temperature balance during the beet growing season. The hydrothermal coefficient (HTC) was calculated according to Selyaninov (1928) [26] as the ratio of precipitation to one-tenth of the sum of active temperatures (mean daily temperatures exceeding 10 °C) [23]. The highest HTC during budding and early flowering (May) was recorded in 2026 (HTC = 2.73), compared with 1.25 in 2025 (Table 1). During flowering time in June, the HTC values were 0.27, 0.22, and 0.44 in 2024, 2025, and 2026, respectively.

The mean daily air temperature in May (budding period of second-year plants) was 17.6 °C in 2024, 20.8 °C in 2025, and 18.1 °C in 2026. These values were 1.3–4.5 °C higher than the long-term average (16.3 °C). In June, air temperatures across all three years also exceeded the climatic norm by 3.3–4.4 °C.

The mean minimum air temperature in May was 12.4 °C in 2024, 14.2 °C in 2025, and 11.1 °C in 2026. The number of nights with temperatures below 10 °C was 2, 5, and 9 in 2024, 2025, and 2026, respectively (Supplementary Table S2).

### 2.4. Assessment of Anther Morphology and Pollen Fertility

Phenotypic characteristics of the anthers were assessed twice within a two-week period. Anthers were visually examined and classified by color and morphology into three categories: (1) yellow, rounded anthers; (2) pale-yellow (dull-yellow) anthers that darkened rapidly and were partially shriveled; and (3) brown anthers. Based on anther dehiscence, flowers were classified into three groups: fully dehiscent, partially dehiscent, and non-dehiscent. These categories were based on the description by Moritani et al. (2013) [27].

The acetocarmine staining method was used to assess pollen fertility during the mass flowering phase. Freshly collected anthers were placed on a slide, dissected with a needle, and stained with a drop of 1% acetocarmine. After 5–10 min [28], they were examined using an MT4300L light microscope (Meiji Techno Co., Ltd., Saitama, Japan) equipped with a Vision digital camera.

Pollen grains that had a regular shape, were intensely stained red, and contained cytoplasm were considered fertile, whereas deformed, shriveled, or weakly stained pollen grains were classified as sterile [4]. Five plants per genotype were selected for pollen fertility assessment and were treated as independent biological replicates [8,29]. The same plants were also used for molecular genotyping, allowing for a direct comparison between pollen fertility phenotypes and the corresponding mitotypes at the individual plant level. A minimum of 15–20 pollen grains were examined microscopically for each plant.

Pollen fertility was calculated separately for each of the five plants as the percentage of fertile pollen grains relative to the total number of pollen grains examined for that plant. Thus, each plant was treated as a biological replicate, while the pollen grains examined within that plant were considered subsamples. The pollen fertility values of the five plants in each genotype were used to calculate the mean and standard deviation (mean ± SD). Pollen fertility was classified according to the following scale: sterile (0–9%), semi-fertile (10–40%), and fertile (41–100%).

### 2.5. Genotyping

The genomic DNA was extracted from young leaves of individual plants using the cetyltrimethylammonium bromide (CTAB) method, following a standard protocol [30]. For each accession, one young leaf was collected from each of five individual plants, and DNA was extracted and analyzed separately. DNA concentration was analyzed with a NanoDrop spectrophotometer (Thermo Fisher Scientific, Waltham, MA, USA), and sample integrity was evaluated through 1% agarose electrophoresis. All DNA samples were adjusted to a final concentration of 100 ng/µL.

Molecular markers for mitochondrial and chloroplast genes were used to differentiate cytoplasm types. The mitochondrial markers TR1–TR3 were amplified using primers described by Nishizawa et al. (2000) [3]. The fragment sizes obtained using the TR1–TR3 markers were interpreted based on previously published data. For the TR1 marker, haplotypes were identified based on the variable number of tandem repeats, following the nomenclature described by Nishizawa et al. (2007) [31] and later refined by Cheng et al. (2009) [8] and Ohgami et al. (2016) [32]. According to this classification, the 410 bp fragment corresponds to 4 copies of the tandem repeat, the 474 bp to 6 copies, and the 700 bp to 13 copies. For the TR2 marker, classification followed the fragments described by Liu et al. [33], with the 362 bp fragment corresponding to 3 repeats, and the 461 bp one to 4 repeats. For the TR3 marker, the 442 bp fragment was recorded as corresponding to 3 repeats, with the 376 bp fragment corresponding to 2 repeats, according to Ohgami et al. [32].

Mitotype (min) classification with TR1–TR3 markers was determined according to Nishizawa et al. (2007) [31].

The chloroplast marker targeted the *petG*-*psbE* intergenic spacer. Sterile sugar beet forms carry InDels (insertions/deletions) in this region that correlate with the CMS phenotype, which is why this locus was chosen. Primers E and G, flanking a conserved 798 bp region common to both cytoplasm types, were used for amplification [10]. The PCR product was digested with the restriction endonuclease *Hin*dIII (Thermo Fisher Scientific, USA) to distinguish between normal (N) and sterile (S, Owen-type) cytoplasms. Digestion was carried out for 4 h at 37 °C with the manufacturer’s supplied buffer. *Hin*dIII digestion of the chloroplast marker produced two restriction fragments, 473 and 325 bp, in the sterile Owen-type cytoplasm (S-type), whereas the 798-bp amplicon remained undigested in the normal fertile cytoplasm (N-type).

The primer sequences, expected fragment sizes for each marker, and PCR conditions are provided in Supplementary Table S3. Oligonucleotides were synthesized by Thermo Fisher Scientific (Pleasanton, CA, USA).

PCR amplification was performed in a final volume of 15 µL, containing 2 µL of genomic DNA (100 ng), 1.5 µL of 10× PCR buffer, 2 µL of MgCl_2_ (2.5 mM), 0.7 µL of dNTP mix (2.5 mM each), 0.5 µL of each primer (1 pmol/µL), 0.5 µL of BSA, and 0.15 µL of Taq polymerase (5 U/µL; Biosan, Novosibirsk, Russia). The amplification reactions were carried out in an Eppendorf Mastercycler (Bertsdorf, Germany). The same PCR cycling profile was used for all mitochondrial TR1–TR4 and chloroplast *petG*-*psbE* markers: initial denaturation at 94 °C for 3 min; 35 cycles of denaturation at 94 °C for 15 s, annealing at 56 °C for 20 s, and extension at 72 °C for 60 s; and a final extension at 72 °C for 5 min. PCR and restriction products were separated by electrophoresis on 8% polyacrylamide gel (AppliChem, Darmstadt, Germany) at 200 V for 75 min. Gels were stained with ethidium bromide (0.5 µg/mL) and documented using a Quantum ST4 UV transilluminator (Vilber-Lourmat, Marne-la-Vallée, France). Fragment sizes were determined by comparison with a 50 bp DNA ladder (Step50 plus, BiolabMix, Novosibirsk, Russia).

## 3. Results

### 3.1. Characterization of Mitochondrial Cytoplasmic Types Using TR1–TR3 Markers

#### 3.1.1. Analysis of the TR1 Mitochondrial Marker

**Figure 1:**
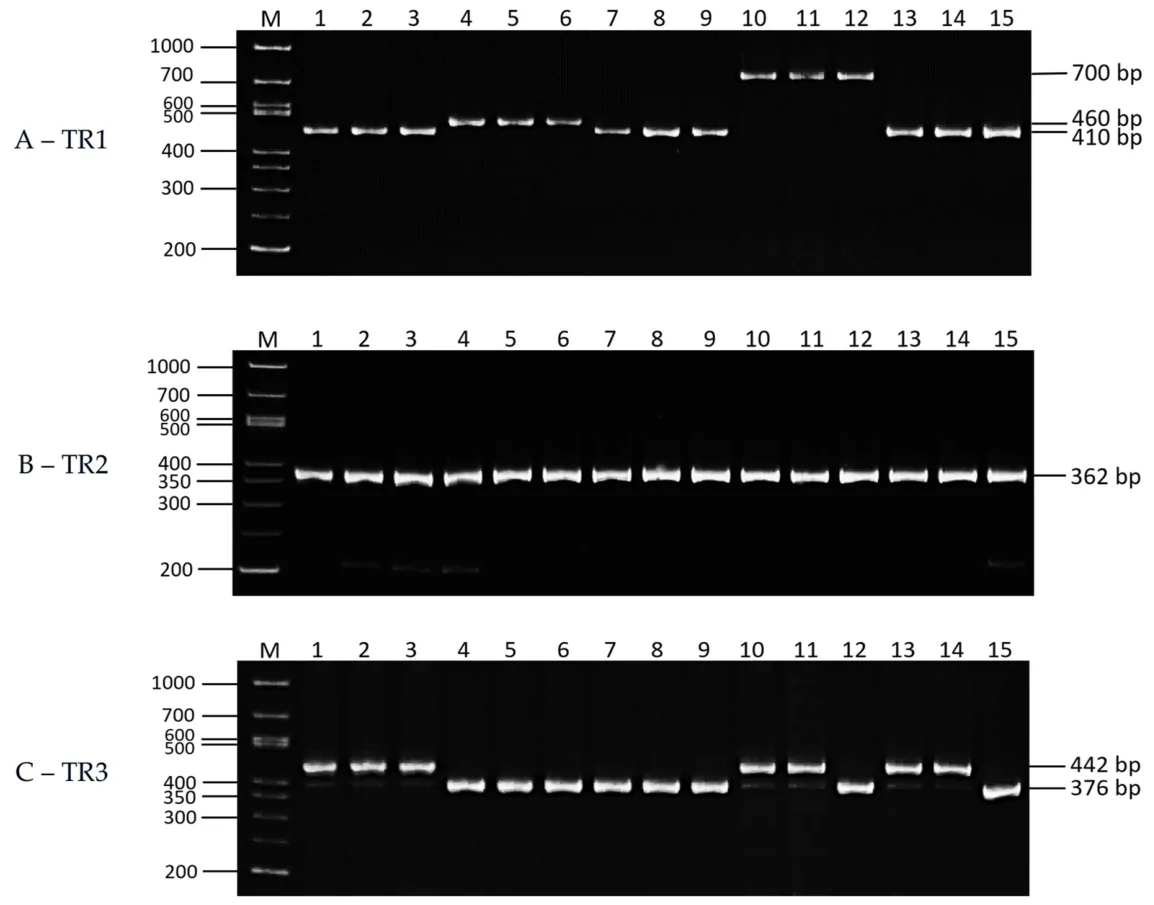
Identification of three individual plants per sugar beet genotype based on alleles of the mitochondrial markers, TR1–TR3. PCR products were separated by 8% polyacrylamide gel electrophoresis. M, Step50 molecular weight marker. (**A**) Marker TR1: Lanes 1–3, reference material PI 594911 (FC 721 CMS) with known S-type cytoplasm (min04). Lanes 4–6, 16956 (*Beta patula* × O-type). Lanes 7–9, MS 3036. Lanes 10–12, PI 632251 (FC 724) with known N-type cytoplasm (min18). Lanes 13–15, ChS-97. (**B**) Marker TR2: Lanes 1–3, 16951 (*Beta maritima* × O-type). Lanes 4–6, MS 2093. Lanes 7–9, ChS-1631. Lanes 10–12, MS 22009. Lanes 13–15, FC 724 O-type. (**C**) Marker TR3: Lanes 1–3, FC 724 O-type. Lanes 4–6, MS 1949. Lanes 7–9, ChS-component. Lanes 10–12, FMS167. Lanes 13–15, MS3037.

PCR amplification with the TR1 marker [3] revealed amplicon length polymorphism across the studied panel of 30 sugar beet accessions. Depending on genotype, three distinct fragments of 410, 460, and 700 bp were detected (Figure 1). Of these, the amplicons of 410 and 700 bp, described earlier [32], corresponded to 4 and 13 tandem repeats, respectively, and these data are present in Supplementary Table S1.

Lines FC 721 CMS (Washington State University, USA) and FMS-1-FD (Florimond Desprez, France) were used as positive CMS controls. Both control lines exhibited an amplicon of approximately 410 bp. A similar fragment was detected in all CMS lines with various origins, from Russia, Belarus, Ukraine, Poland, and Kazakhstan (Figure 1A and Supplementary Table S1).

In the O-type control lines FC 724 and RF 2093, a 700 bp amplicon was detected, corresponding to 13 tandem repeats. This fragment is typical for normal cytoplasm (Normal-1, mitotype min18) according to the classification of Nishizawa et al. (2007) [31,32]. Genetic polymorphism for this marker was detected in eight breeding lines based on an analysis of five plants per line: ChS-1631, ChS-1638, FMS167, FMS173, MS9047, MS7, MS3037, and MS Aisholpan. In addition to the predominant 410 bp allele, an amplicon of approximately 700 bp, corresponding to 13 tandem repeats, was detected in individual plants.

An amplicon of approximately 460 bp was detected in alloplasmic lines from Ukraine derived from introgression of sterile cytoplasm from wild species *B. maritima* and *B. patula* [34]. This amplicon was found exclusively in Ukrainian alloplasmic breeding lines. According to Ohgami et al. (2016) [32], the length of this fragment corresponds to five tandem repeats.

#### 3.1.2. Analysis of the TR2 Mitochondrial Marker

Analysis of 30 sugar beet breeding lines using the TR2 marker [3] revealed a 362 bp amplicon in all examined lines (Figure 1B). Identical PCR profiles were found in all breeding lines using this marker regardless of their origin. This finding indicated an absence of genetic polymorphism in the studied genotypes using the TR2 marker. Liu et al. (2017) [33] classified the 362 bp amplicon size as three tandem repeats (Table S1).

#### 3.1.3. Analysis of the TR3 Mitochondrial Marker

The application of the TR3 marker [3] showed two amplified fragments of 376 and 442 bp in 30 studied sugar beet breeding lines (Figure 1C). The 376 bp amplicon was detected in 22 genotypes and in CMS-positive controls, FC 721 CMS, and FMS-1-FD. According to Ohgami et al. (2016) [32], this amplicon corresponded to two tandem repeats, while three repeats resulted in a 442 bp PCR amplicon in the O-type positive controls, FC 724 and RF 2093 (Table S1).

Incomplete uniformity for the TR3 marker, indicating non-homogeneous genotypes, was detected in five plants within each of eight breeding lines studied, including ChS-1631, ChS-1638, FMS167, FMS173, MS9047, MS7, MS3037, and MS Aisholpan. Individual plants within these lines exhibited both amplicon sizes, 376 and 442 bp (Figure 1C).

#### 3.1.4. Mitotype Classification

Data on the number of tandem repeats at the mitochondrial minisatellite loci TR1–TR3 are presented in Table 1. Based on the repeat structure at these loci, sugar beet mitotypes were determined according to the classification systems of Nishizawa et al. (2007) [31] and Cheng et al. (2009) [8]. Sugar beet genotypes have four tandem repeats at TR1, three at TR2, and two at TR3, forming the repeat combination code ‘4,3,2’, and these were classified as mitotype min04. The O-type breeding lines, used as positive controls, belong to mitotype min18 with the repeat combination code ‘13,3,3’, corresponding to the normal cytoplasm type, Normal-1.

Ukrainian alloplasmic sugar beet breeding lines with introgressed cytoplasm from wild species *B. maritima* and *B. patula* were classified as mitotype min06 with the repeat combination code ‘5,3,2’. This mitotype is regarded as one of the key sources of CMS originating from a wild gene pool [9,34] (Table 1).

### 3.2. CAPS Marker Analysis in the petG-psbE Chloroplast Region

**Figure 2:**
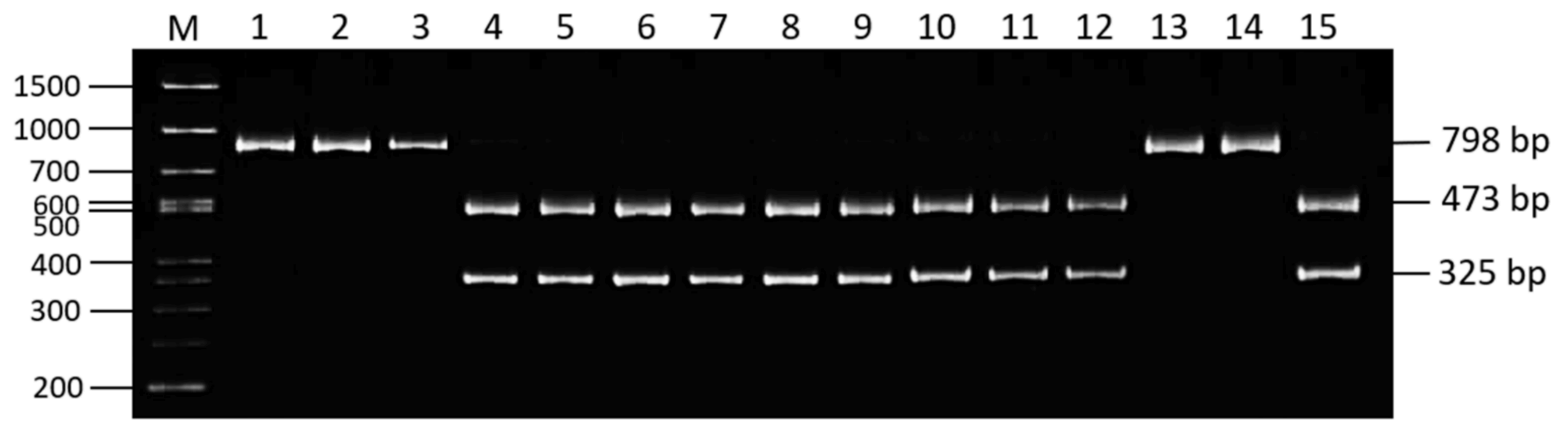
Identification of the *petG*-*psbE* chloroplast region in three individual plants of sugar beet genotypes after digestion with *Hin*dIII. Digested PCR fragments were separated on 8% polyacrylamide gel. M, Step50 molecular weight marker. Lanes 1–3, reference genotype PI 632251 (FC 724) with known N-type cytoplasm. Lanes 4–6, PI 594911 (FC 721 CMS) with known S-type Owen cytoplasm. Lanes 7–9, MS 2093. Lanes 10–12, 16951 (*B. maritima* × O-type). Lanes 13–14, MS 7.

Primers G and E, specific to the *petG*-*psbE* chloroplast region [10], were used to confirm the presence of Owen-type cytoplasm in the examined sugar beet genotypes. The resulting PCR amplicons of 798 bp in all samples were digested with *Hin*dIII endonuclease, and the size of the digested fragments distinguished normal and Owen-type cytoplasm. In the O-type lines, FC 724 and RF 2093, used as positive controls, the amplicon remained unchanged after digestion with a single 798 bp fragment, whereas most of the 22 remaining genotypes showed two smaller fragments of 473 and 325 bp. Notably, alloplasmic lines with min06 mitotype also displayed Owen-type cytoplasm (Figure 2, Table 1).

Eight genotypes were not homogeneous, with five plants analyzed per genotype, including ChS1631, ChS1638, MS 9047, FMS167, FMS 173, MS7, MS3037, and MS Aisholpan. Some of them produced the expected two bands of restricted fragments, whereas others showed only a single band of 798 bp. The presence of the single band indicates the absence of a *Hin*dIII restriction site in the *petG*-*psbE* chloroplast region, a feature indicator for normal (N) cytoplasm. The same pattern was observed in the genotype ChS-component, although it was assigned to the *min04* mitotype.

### 3.3. Evaluation of Anther Morphology and Pollen Fertility

Thirty sugar beet breeding accessions and four control genotypes were evaluated over three consecutive growing seasons (2024–2026) in southeastern Kazakhstan. During the flowering period, anther color, dehiscence, and pollen fertility were assessed. Results are presented in Table 2.

**Figure 3:**
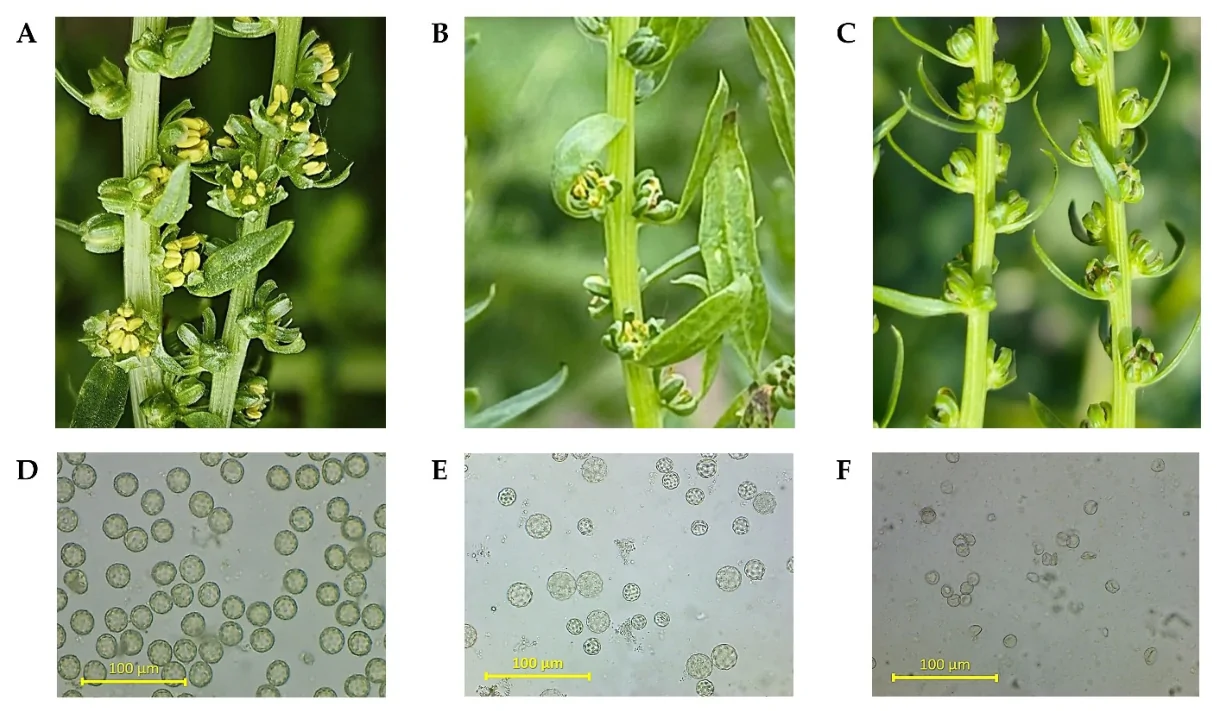
Sugar beet shoots with flowers showing differing degrees of male fertility and anther content. (**A**) Completely fertile flower with well-developed and non-dehisced anthers. (**B**) Flowers with yellow, partially dehiscent anthers. (**C**) Male-sterile flower with pale, later turning brown, shriveled anthers. (**D**) Fertile pollen. (**E**) Semi-fertile pollen. (**F**) Sterile pollen. Scale bars in (**D**–**F**) = 100 µm.

Three anther colors were identified during visual observations among the 34 genotypes examined: yellow, pale yellow, and brown (Figure 3C). Across all three years, yellow anthers were predominant under the growing conditions in southeastern Kazakhstan (Figure 3B). On average, yellow anthers were observed annually in 72.6% of the 34 genotypes evaluated, including 30 sugar beet breeding accessions and 4 control genotypes. Pale yellow and brown anthers occurred considerably less frequently and were observed in 16.7% and 10.9% of tested plants, respectively. The distribution of genotypes across anther color categories remained stable over the three-year period (χ^2^ = 0.697, df = 4, *p* = 0.954), indicating an absence of statistically significant differences for this trait (Table 2).

In addition to color, anther dehiscence varied among the three years of study. Partially dehiscent anthers predominated in 2024 and 2025, whereas the occurrence of non-dehiscent anthers increased in 2026. The chi-squared test confirmed statistically significant differences in anther dehiscence among years (χ^2^ = 11.1, df = 4, *p* = 0.026) in the studied sugar beet breeding lines (Table 2).

A similar trend was observed in the pollen fertility evaluation. Semi-fertile genotypes predominated in 2024 and 2025 (Figure 3E), while the highest occurrence of fertile genotypes was recorded in 2025. The occurrence of completely sterile genotypes increased in 2026 (Figure 3F). Chi-squared analysis confirmed statistically significant differences in pollen fertility between years (χ^2^ = 9.97, df = 4, *p* = 0.041) (Table 2). Accessions FC 721 CMS, FMS-1-FD, and ChS-komponent exhibited stable pollen sterility. Thus, anther color remained stable across the three studied years, whereas dehiscence and pollen fertility exhibited statistically significant variability between years (Table 1).

Among the cytoplasmically heterogeneous breeding lines carrying both min04 and min18 mitotypes, pollen fertility phenotypes differed between the two mitotypes. Plants carrying min18 predominantly exhibited the fertile phenotype (85.7%), whereas min04 plants were predominantly semi-fertile or sterile, with only 20.0% exhibiting the fertile phenotype.

## 4. Discussion

The present study revealed cytoplasmic diversity and variation in CMS phenotypic expression among the sugar beet breeding lines of diverse origins.

### 4.1. Polymorphism of TR1–TR3 Markers and Mitotype Identification

The TR1 marker was the most polymorphic among the mitochondrial loci studied, detecting three amplicons of 410, 460, and 700 bp. The high level of TR1 variability was consistent with previously published data, where the number of alleles ranged from four [32] to six [9]. This confirms the efficiency of TR1 as a highly polymorphic marker in the sugar beet mitochondrial genome.

In the current study, intra-line heterogeneity at the TR1 locus was detected in eight breeding lines, consistent with observations by Cheng et al. (2011) [9], who reported different mitochondrial haplotypes among individual sugar beet plants. The presence of different mitochondrial variants within the same breeding line may indicate incomplete cytoplasmic uniformity or reflect specific aspects of line maintenance and propagation. These findings underscore the importance of molecular monitoring at the individual plant level to maintain cytoplasmic uniformity in CMS breeding lines.

In contrast, the TR2 marker was completely monomorphic. All 30 accessions showed a single 362 bp amplicon. This finding was consistent with previous reports about the highly conserved locus TR2, typically represented by three repeated copies in most sugar beet genotypes [3,9,34].

Analysis of the TR3 mitochondrial marker revealed two stable allelic variants, 376 and 442 bp, corresponding to two and three tandem repeats [32]. These results are consistent with previously published ones [8,9]. The predominant allele of 376 bp was detected in 22 breeding lines, whereas the 442 bp amplicon was typical for O-type cytoplasm. Intra-line heterogeneity for TR3 was observed to be the same in eight accessions that showed heterogeneity at the TR1 locus. In each accession, plants with an alternative cytoplasmic type were found, consistent with the results observed for TR1. Marker TR3, therefore, showed moderate variability, and it can potentially be used together with TR1 for the identification of cytoplasm gene structure in sugar beet germplasm collections more comprehensively [32].

The TR1–TR3 results confirm that the mitochondrial minisatellite marker system was highly informative for distinguishing cytoplasm types. The identified mitotypes, min04 and min18, correspond to S-type (CMS) and Normal-1 cytoplasm, respectively. This is in agreement with previously established classifications and confirms the accuracy of cytoplasmic type identification in the studied breeding lines. Importantly, the detection of different mitotypes within individual breeding lines provided an opportunity to examine whether this cytoplasmic heterogeneity was associated with pollen fertility variation.

The comparison of individual plants within cytoplasmically heterogeneous breeding lines revealed a clear association between mitotype and pollen fertility. Most plants carrying min18 were fertile, whereas plants carrying min04 were mainly semi-fertile or sterile. This pattern suggests that the min04 mitotype is associated with a stronger expression of the male-sterile phenotype. However, the different fertility phenotypes observed among min04 plants indicate that mitotype alone cannot fully explain CMS expression. Nuclear genetic background and environmental conditions may also influence this trait.

### 4.2. Origin and Distribution of Mitotype ‘min06’

The alloplasmic breeding lines developed using *B. maritima* and *B. patula* are of particular interest. These lines were classified as mitotype *min06*, with a repeat combination code of ‘5,3,2’. This mitotype has previously been considered one of the probable sources of CMS in the evolutionary and breeding history of sugar beet [9].

The detection of mitotype min06 in the studied lines confirms the introgression of genetic material from wild sugar beet species. According to the literature, this mitotype was associated with *B. maritima* (sea beet) and considered one of the cytoplasm types derived from wild populations [6,9]. It has previously been shown that CMS sugar beet breeding line I-12CMS(3) also carries mitotype min06, which originated from wild beet species native to Pakistan. However, the distribution of min06 was not limited to Pakistan. This mitotype has also been reported in various regions of Eastern Europe, Greece, Turkey, Japan, and China [9]. In contrast to Owen-type cytoplasm (min04), which is widely used in hybrid breeding, mitotype min06 represents an alternative and ‘wild’ type of CMS associated with a very different E-type CMS, as described earlier [35]. Its presence in the collection indicates the preservation of cytoplasmic genome diversity and highlights the potential value of these breeding lines for broadening the sugar beet germplasm gene pool.

### 4.3. Analysis of petG-psbE Chloroplast Marker

The analysis of the CAPS marker in the chloroplast *petG*-*psbE* region with *Hin*dIII endonuclease digestion demonstrated that the majority of the studied CMS lines carried the additional restriction site associated with Owen-type cytoplasm. However, the alternative undigested 798 bp pattern was detected in the ChS-component genotype and in individual plants of several heterogeneous accessions, indicating intra-line variation in the chloroplast marker profile. Together with the mitochondrial marker analysis, these results provided complementary information on the cytoplasmic composition and revealed cytoplasmic heterogeneity within part of the studied breeding material.

The most notable finding in the current study was the combination of mitotype min06, a mitochondrial marker associated with wild sterile cytoplasm, and a chloroplast pattern of Owen-type cytoplasm in alloplasmic breeding lines. Initially, this combination seems contradictory. However, it is consistent with existing evidence that mitochondrial and chloroplast genomes, despite being co-inherited maternally, may exhibit different patterns of molecular variation and evolutionary differentiation.

The CMS trait in sugar beet is determined by rearrangements in mitochondrial DNA, whereas the chloroplast genome is not directly involved in the induction of sterility. It was demonstrated earlier that mitochondrial and chloroplast genomes of breeding lines with diverse sterile cytoplasm can differ in their degree of variability depending on their distinct introgression origins [36,37]. Thus, the discordance between the mitochondrial and chloroplast marker results may reflect the complex history of the two organellar genomes rather than a single cytoplasmic origin. One possible explanation for the observed min06/Owen-type combination is the differential retention or sorting of mitochondrial and chloroplast genomes during the alloplasmic material development. This process may have resulted in an asynchronous contribution of the two organellar components. However, since the studied lines were developed through sexual hybridization followed by repeated backcrossing [34], and both organellar genomes are predominantly maternally inherited, the present marker data do not allow this mechanism to be confirmed.

Somatic hybridization or protoplast fusion may also generate combinations in which chloroplasts and mitochondria have different parental origins, as demonstrated in experimentally produced somatic hybrids [38]. However, this mechanism is unlikely to explain the present material because the alloplasmic lines were developed by conventional hybridization and successive backcrossing rather than by somatic hybridization or protoplast fusion [34].

The observed combination of mitotype *min06* and the *petG*-*psbE*/*Hin*dIII marker for Owen-type cytoplasm therefore indicates an unusual combination of mitochondrial and chloroplast markers in the studied alloplasmic sugar beet lines. This combination may have been present in the original wild cytoplasmic donors or may reflect differential histories of the two organellar genomes during the development of the alloplasmic material. Analysis of the original *B. maritima* and *B. patula* cytoplasmic donors and parental sugar beet lines with both mitochondrial and chloroplast markers would help clarify the origin of this combination.

### 4.4. Effect of Hydrothermal Conditions on the Phenotypic Expression of CMS

According to published data, CMS lines are typically characterized by pale-yellow, whitish, or brownish anther colors, attributed to impaired anther tissue development and premature tapetal degeneration [39,40]. In contrast, in the present study, yellow anther color was observed in the majority of the studied CMS lines (72.6% on average), whereas pale-yellow forms were less common. Despite variation in environmental conditions across the three growing seasons, the distribution of breeding lines by anther color remained stable and showed no statistically significant differences between years. It should be noted that this trait is not only determined by cytoplasm type; rather, it may also reflect interactions with the nuclear genome, which was not analyzed in the current study.

Anther dehiscence and pollen fertility exhibited pronounced variability between years. The most pronounced deviations were observed in 2026, when the number of breeding lines with non-dehiscent anthers increased, accompanied by a concurrent rise in the proportion of fully sterile plants. This growing season was characterized by the highest hydrothermal coefficient (HTC) during the shoot growing and early flowering periods in May 2026 (HTC = 2.73) compared to HTC = 1.25 in May 2025.

Differences in temperature were also noted between years. In the warmer 2025 season, the highest number of breeding lines with fertile pollen was recorded. In contrast, the cooler and wetter conditions of 2026 were associated with an increased proportion of sterile plants. These findings indicate that interannual variation in CMS-related traits occurred under contrasting hydrothermal conditions. However, the three-year observational design does not allow for the effects of temperature, precipitation, and genotype × environment interactions to be separated statistically. Therefore, the observed associations should be interpreted as interannual patterns rather than evidence of a direct causal effect of individual meteorological factors or HTC on CMS expression.

This trend is consistent with existing evidence about the high sensitivity of anther developmental processes to abiotic stress, including tapetal differentiation and microsporogenesis. The degree of CMS phenotype expression is determined by multiple factors, including the type of cytoplasm, interactions between the mitochondrial and nuclear genomes, and environmental conditions. 

Taken together, these three-year observations demonstrate that molecular identification of CMS breeding lines can vary substantially in genotypes with stable or unstable sterility depending on growing conditions. These results highlight the need for an integrated approach that combines molecular identification of cytoplasm with multi-year evaluation of phenotypes under different hydrothermal conditions. This is essential for the selection of stable parental genotypes for the breeding of sugar beet hybrids.

## 5. Conclusions

Combined mitochondrial and chloroplast marker analysis revealed substantial cytoplasmic diversity in the studied sugar beet breeding material, with min04 representing the predominant CMS mitotype and min06 characterizing the alloplasmic lines derived from *Beta maritima* and *B. patula*. Individual plant genotyping revealed cytoplasmic heterogeneity within several breeding lines, emphasizing the importance of molecular monitoring during CMS line maintenance. The unusual combination of the min06 mitochondrial mitotype with the Owen-type chloroplast CAPS marker indicates a complex organellar composition whose origin requires further investigation in terms of the parental cytoplasmic donors. Multi-year phenotypic evaluation demonstrated significant interannual variation in anther dehiscence and pollen fertility, coinciding with differences in hydrothermal conditions during flowering. Overall, the integration of organellar marker analysis with multi-year phenotyping provides an effective approach for characterizing CMS sources, monitoring cytoplasmic uniformity, and evaluating the stability of male sterility in sugar beet breeding material.

## Figures and Tables

**Table 1:** Cytoplasm types identified by mitochondrial minisatellite and chloroplast *petG*-*psbE* CAPS markers and pollen fertility phenotypes of 30 sugar beet breeding accessions and four control genotypes.

Accession	TR1	TR2	TR3	Mitotype	*petG*-*psbE*	Fertility Phenotype (2024–2026)
FC 721 CMS	4	3	2	min04	Present	Stable sterile
FMS-1-FD	4	3	2	min04	Present	Stable sterile
16951 (*Beta maritima* × O-type)	5	3	2	min06	Present	Stable semi-fertile
16952 (*Beta patula* × O-type №1)	5	3	2	min06	Present	Variable
16954 (*Beta maritima* × O-type)	5	3	2	min06	Present	Variable
16956 (*Beta patula* × O-type)	5	3	2	min06	Present	Variable
ChS-97	4	3	2	min04	Present	Variable
ChS-1631	4/13	3	2/3	min04/min18	Present/Absent	Variable
ChS-1611	4	3	2	min04	Present	Stable semi-fertile
ChS-1633	4	3	2	min04	Present	Stable semi-fertile
ChS-1638	4/13	3	2/3	min04/min18	Present/Absent	Variable
ChS-komponent	4	3	2	min04	Absent	Stable sterile
MS Perla	4	3	2	min04	Present	Stable semi-fertile
MS 1B	4	3	2	min04	Present	Variable
MS 7	4/13	3	2/3	min04/min18	Present/Absent	Stable semi-fertile
MS 95	4	3	2	min04	Present	Stable semi-fertile
MS 94AP	4	3	2	min04	Present	Variable
MS 1949	4	3	2	min04	Present	Variable
MS 2093	4	3	2	min04	Present	Variable
MS 2113	4	3	2	min04	Present	Variable
MS 9047	4/13	3	2/3	min04/min18	Present	Variable
MS22009	4	3	2	min04	Present	Variable
MS22014	4	3	2	min04	Present	Stable semi-fertile
FMS 162	4	3	2	min04	Present	Stable semi-fertile
FMS 167	4/13	3	2/3	min06/min18	Present/Absent	Variable
FMS 173	4/13	3	2/3	min04/min18	Present/Absent	Variable
FMS 183	4	3	2	min04	Present	Stable semi-fertile
MS1	4	3	2	min04	Present	Stable semi-fertile
MS Aisholpan	4/13	3	2	min04/min18	Present/Absent	Variable
MS 3035	4	3	2	min04	Present	Variable
MS 3036	4	3	2	min04	Present	Variable
MS 3037	4/13	3	2/3	min04/min18	Present/Absent	Stable semi-fertile
FC 724 O-type	13	3	3	min18	Absent	Stable fertile
RF 2093	13	3	3	min18	Absent	Stable fertile

**Table 2:** Phenotypic characterization of anthers in 30 sugar beet breeding accessions and four control genotypes evaluated in 2024–2026, along with results of the chi-squared (χ^2^) test.

Morphological Characteristic	Categories	Occurrence			Mean	Chi-Squared Tests		
		2024	2025	2026		χ^2^	df	*p*
Anther color	Brown	4	3	4	3.7 ± 0.5	0.697	4	0.954
	Pale-yellow	5	7	5	5.7 ± 0.9			
	Yellow	25	24	25	24.7 ± 0.5			
	Total	34	34	34				
Anther dehiscence	Full	6	8	4	6.0 ± 1.6	11.1	4	0.026
	Non-full	23	21	15	19.7 ± 3.4			
	None	5	5	15	8.3 ± 4.7			
	Total	34	34	34				
Pollen fertility	Fertile	7	13	3	7.7 ± 4.1	9.97	4	0.041
	Semi-fertile	24	17	24	21.7 ± 3.3			
	Sterile	3	4	7	4.7 ± 1.7			
	Total	34	34	34				

## Data Availability

The original contributions presented in this study are included in this article and its Supplementary Materials. Further inquiries can be directed to the corresponding author.

## Supplementary Materials

The following supporting information can be downloaded at https://www.mdpi.com/article/10.3390/life16091413/s1, Supplementary Material S1: Table S1. Origin, mitochondrial (TR1–TR3) and chloroplast (*petG-psbE*) marker profiles, and anther morphological characteristics of the studied sugar beet genotypes; Supplementary Material S2: Table S2. Meteorological data (air temperature and precipitation) and hydrothermal conditions at the KRIAPG field trials during 2024–2026; Table S3. DNA markers and PCR amplification conditions used for sugar beet genotyping.

## Abbreviations

The following abbreviations are used in this manuscript:

CMS	Cytoplasmic male sterility
CAPS	Cleaved amplified polymorphic sequence
KazRIAPG	Kazakh Research Institute of Agriculture and Plant Growing
VNTR	Variable number tandem repeat
Min	Mitotype
PCR	Polymerase chain reaction
