# Supplementary material for: Cytoplasmic Male Sterility in Sugar Beet: Mitochondrial Minisatellite and Chloroplast CAPS Markers and Field Phenotype Stability Study
Source: Life (Basel). 2026 Aug 26;16(9):1413. doi: 10.3390/life16091413 (PMC13608664; doi:10.3390/life16091413)
Supplement: Supplementary file 1 [file life-16-01413-s001.zip › Supplementary Material S2.pdf]

Supplementary Materials to the paper

Cytoplasmic Male Sterility in Sugar Beet: Mitochondrial Minisatellite and Chloroplast CAPS Markers, and Field Phenotype Stability Study

Raushan Yerzhebayeva, Assel Jenisbayeva, Alfiya Abekova, Natalia Kovalchuk , Sholpan Bastaubayeva , Aigul Amangeldiyeva and Laila Tabynbayeva

Table S2. Meteorological data (air temperature and precipitation) and hydrothermal conditions at the KRIAPG field trials during 2024–2026.

Month	Year	Air temperature, °C					Precipitation, mm		HTC
		Mean max. temperature	Mean min. temperature	Number of cold nights (<10 °C)	Mean monthly temperature	Multi-year average	Monthly	Multi-year average	
March	2024	15.7	6.6	30	5.4	-4.4	135.5	65.6	-
April		17.8	7.9	22	12.8	10.9	111.3	110.6	2.80
May		23.6	12.4	5	17.6	16.3	121.2	98.4	2.22
June		30.4	18.8	0	24.5	21.2	19.7	59.9	0.27
July		31.3	19.1	0	25.0	23.7	85.2	56.9	1.1
March	2025	11.9	1.3	28	6.2	2.8	76.8	64.7	-
April		21.5	9.8	15	15.7	10.9	57.2	110.6	1.18
May		27.0	14.2	2	20.8	16.3	80.4	98.0	1.25
June		31.4	19.6	0	25.6	21.2	17.1	59.9	0.22
July		34.2	21.1	0	27.7	23.7	9.6	56.9	0.11
March	2026	12.0	0.8	30	4.9	2.8	51.2	64.7	-
April		21.1	9.2	13	15.4	10.9	107.2	110.6	2.25
May		23.2	11.1	9	18.1	16.3	153.2	98.4	2.73
June		30.9	20.1	0	25.3	21.2	33.3	59.9	0.44

Table S3. DNA markers and PCR amplification conditions used for sugar beet genotyping

Marker	Sequence of Primer (5′→3′)	Annealing T (°C)	Expected PCR product / diagnostic fragments (bp)	References
TR1	AGAACTTCGATAGGCGAGAGG<br>GCAATTTTCAGGGCATGAACC	56	410, 474, 700 etc.	[3]
TR2	TTAATTGCGAGACCGGAGGC<br>GAGCTTGCTCGCAGCTTATG	56	362, 461 etc.	
TR3	AGATCCAAACAGAGGGACTG<br>CGGATCACCCTATTCATTTG	56	376, 442 etc.	
E/G	TAAAGGAAAAGATAAGAG<br>TGGGAATTGCCCATGAAT	56	798; after *Hind*III: 473 + 325 (S-type)	[10]
